# FLIR vs SEEK thermal cameras in biomedicine: comparative diagnosis through infrared thermography

**DOI:** 10.1186/s12859-020-3355-7

**Published:** 2020-03-11

**Authors:** Ayca Kirimtat, Ondrej Krejcar, Ali Selamat, Enrique Herrera-Viedma

**Affiliations:** 1 Faculty of Informatics and Management, Center for Basic and Applied Research, University of Hradec Kralove, Rokitanskeho 62, 500 03 Hradec Kralove, Czech Republic; 2 Malaysia Japan International Institute of Technology (MJIIT), Universiti Teknologi Malaysia Kuala Lumpur, Jalan Sultan Yahya Petra, Kuala Lumpur, 54100 Malaysia; 3 Digital Cities Research Institute, Multimedia University, Persiaran Multimedia, Cyberjaya, 63100 Malaysia; 4 Media and Games Center of Excellence (MagicX) Universiti Teknologi Malaysia & School of Computing, Faculty of Engineering, Universiti Teknologi Malaysia, Skudai, 81310 Johor Malaysia; 5 Andalusian Research Institute in Data Science and Computational Intelligence, University of Granada, 18071 Granada, Spain; 6 Department of Electrical and Computer Engineering, Faculty of Engineering, King Abdulaziz University, Jeddah, Saudi Arabia

**Keywords:** Comparative diagnosis, Infrared thermography, Biomedicine, Infrared camera, FLIR, SEEK

## Abstract

**Background:**

In biomedicine, infrared thermography is the most promising technique among other conventional methods for revealing the differences in skin temperature, resulting from the irregular temperature dispersion, which is the significant signaling of diseases and disorders in human body. Given the process of detecting emitted thermal radiation of human body temperature by infrared imaging, we, in this study, present the current utility of thermal camera models namely FLIR and SEEK in biomedical applications as an extension of our previous article.

**Results:**

The most significant result is the differences between image qualities of the thermograms captured by thermal camera models. In other words, the image quality of the thermal images in FLIR One is higher than SEEK Compact PRO. However, the thermal images of FLIR One are noisier than SEEK Compact PRO since the thermal resolution of FLIR One is 160 × 120 while it is 320 × 240 in SEEK Compact PRO.

**Conclusion:**

Detecting and revealing the inhomogeneous temperature distribution on the injured toe of the subject, we, in this paper, analyzed the imaging results of two different smartphone-based thermal camera models by making comparison among various thermograms. Utilizing the feasibility of the proposed method for faster and comparative diagnosis in biomedical problems is the main contribution of this study.

## Background

As a few degree change in the normal temperature of human body indicates possible illnesses [1, 2] thermoregulation process controls this temperature change physiologically [1, 3]. Thus, thermometers, which measure the temperature change of human body below 36.3 °C and above 37.5 °C, were found around seventeenth century [4, 5]. When William Herschel first discovered the infrared radiation, and recorded the first thermal image by his son, new opportunities were provided in the field of temperature measurements [4].

As known, the objects and the subjects (i. e. human body) with the temperature above absolute zero emit the electromagnetic radiation, and this is known as infrared or thermal radiation between the wavelength 0.75–1000 μm [1]. Since, it has a diagnostic importance for human body; we first used the “infrared thermography” term especially in medical sciences in 1960 [1, 5]. Moreover, infrared thermography provide us to recognize defects or abnormal thermal patterns on skin surfaces or subsurfaces [1]. In order to interpret thermal patterns and abnormalities, thermal images or thermograms are created through IR cameras.

IR cameras measure the emitted thermal radiation, a function of surface temperature, on any surface and turn this radiation into thermal images under various color formats available in the camera. Moreover, the IR cameras calculate and reveal inhomogeneous temperature differences on the surfaces of objects or subjects [6]. The recent IR cameras in the industries and markets are the third-generation thermal cameras, which use FPA (focal plane array) and consist of photon (cooled) and thermal detectors (uncooled) together [7]. We generally use thermal cameras with cooled detectors for scientific or military purposes, while we use the cameras with uncooled detectors for transportation, fire safety, building or biomedical applications. However, for the purpose of biomedical diagnosis, FLIR and SEEK infrared camera models with thermal detectors are used. Based on our literature review on biomedical problems, FLIR type infrared cameras are used in the detections more than SEEK type infrared cameras, since they have FPA detectors, which work with thermal detectors.

FLIR camera models conceive temperature differences under 0.02 °C (20 mK) by offering quantitative and in-depth measurements to detect the problems in the deepest body or skin in biomedicine. By virtue of high potential detection capacity of FLIR camera models in biomedical applications, medical researchers reach more explanatory and accurate results [8]. However, since SEEK camera models with thermal detectors which are not satisfying enough to record thermal images in biomedical researches, these camera models are generally used for building diagnostics, outdoors, firefighting and commercial trades. The main reason for this situation is that SEEK camera models are created in order to straightforwardly explore heat sources by smartphone-based applications and these applications could turn a smartphone into a thermal camera. Another reason for preference of SEEK camera models is that they are more cost-efficient than other thermal camera models [9]. In Table 1 the overall differences and similarities of two particular infrared camera models are briefly summarized.

**Table 1:** The particular FLIR vs SEEK model infrared cameras

	FLIR infrared camera	SEEK infrared camera
Application area	Buildings, biomedicine, industry, lab tests	Buildings, outdoors, commercial, firefighting
Detector types	Thermal and photon detectors	Thermal detector
Generation	3rd generation	3rd generation
Infrared spectrums	7–14 μm	7–14 μm
Portability	Standalone and smartphone-based compact	Smartphone-based compact
Measurement type	Quantitative and qualitative	Qualitative

### Related works on FLIR and SEEK in biomedicine

On the purpose of revealing inhomogeneous temperature distribution in human body, skin and organs in biomedicine, the use of IR thermography is passive and non-invasive technique. We mostly use IR thermography for the detection in body shells rather than body structure in biomedicine. Moreover, when a medical problem is further detected by the support of image processing methods, accurate and huge data acquisition is quite achievable. One of the most important criteria for preference of IR thermography methods is no radiation exposure of patients [10]. According to our literature survey on biomedical applications of IR thermography, various experiments could be found through FLIR and SEEK infrared camera models. Table 2 briefly explained these articles with methods, application and thermal camera types.

**Table 2:** Related works on biomedical applications of IR thermography

Authors	Method	Medical Problem Type	Infrared Camera Model
*This paper*	*IRT*	*Injured toe of the subject 35/M*	*FLIR One, SEEK Compact PRO*
Habek et al. (2018)	IRT	Detection of brown adipose tissue	FLIR T-650sc
Silva et al. (2018)	IRT	Plantar Surface Temperature in Diabetes Mellitus	FLIR E60
Sarigoz et al. (2018)	IRT	Diagnosis of breast mass	FLIR ThermaCAM E45
Contreras et al. (2017)	IRT	Thermal changes in diabetic foot	FLIR E60
Estal et al. (2017)	IRT	Investigation of thermal profile	FLIR T335
Alpar and Krejcar (2017)	IRT	Hand thermography	FLIR One
Alpar and Krejcar (2017)	IRT	Hand vein estimation	FLIR One
Silva et al. (2016)	IRT	Breast cancer	FLIR SC620
Peleki and Silva (2016)	IRT	Detection of Acute Limb Ischaemia	SEEK Thermal Compact XR
Oliveira et. el. (2016)	IRT	Diagnosis of sprained ankle injuries	FLIR E60 SC
Chudecka and Lubkowska (2015)	IRT	Thermal map of body surface	ThermaCAM SC500
Queseda et al. (2015)	IRT	Skin temperature, muscle activation	FLIR E60
Queseda et al. (2015)	IRT	Skin temperature during cycling	FLIR E60
Contreras et al. (2015)	IRT	Early diagnosis of diabetic foot	FLIR E60
Araujo et al. (2014)	IRT	Breast cancer	FLIR S45
Fournet et al. (2013)	IRT	Body mapping of females and males	ThermaCAM B2, FLIR
Bernard et al. (2013)	IRT	Hand surface temperature detection	FLIR B200

Fournet et al. [11] investigated regional skin temperature of male and female bodies running in the cold through body-mapping methodology. In this study, different thermoregularity and perceptual variables were also investigated. Whole body temperature was recorded using FLIR ThermaCAM B2 infrared camera. As a result, the authors stated that due to fat patterning in their body, males and females had different thermographic body maps.

In the study of Bernard et al. [12], it was determined that measurement results of the hand thermography would be affected or not by emissivity values of various substances such as ultrasound gel, ointment, disinfection, etc. They recorded all thermograms with the infrared camera FLIR B200 in the experiments. According to the experimental results, the surface temperature of the subject is more or less affected by the various substances, thus the authors stated that these substances would be taken into consideration while conducting thermographic surveys.

As breast cancer is one major diseases for women, Araujo et al. [13] studied symbolic data analysis (SDA) framework for evaluating the breast abnormalities by detecting breast cancer at the same time. In order to classify breast abnormalities, the authors proposed three staged process, which are acquisition, segmentation and morphological processing of the thermographic images. In the image acquisition stage, all thermograms of a patient group were captured by FLIR S45 infrared camera in a hospital in Brazil. The authors transformed each thermal image into a temperature matrix to extract breast areas of the thermograms. What they concluded from this research is that thermography is one of the complementary method for breast screening before mammography evaluation.

Contreras et al. [14] studied the automatic classification of thermal patterns for thermographic images of a diabetic foot to support early diagnosis. 44 different thermograms were captured in a room at controlled temperature in the research. The authors captured the images with the infrared camera FLIR E60 with a thermogram resolution of 320 × 240 pixels. It was also stated in the article that detecting abnormal differences in temperature could be important method for early diagnosis of risky areas on the foot by IR thermography.

Another study by Quesada et al. [15] was about comparison between infrared thermography and thermal contact sensors for the measurement of skin temperature during cycling. The authors conducted an instrumented test with 14 cyclists by comparing the presented methodologies when simulating heat exchange in dry and wet conditions. The thermograms were recorded with the infrared camera FLIR E60 an infrared resolution of 320 × 240 pixels. According to comparable results of the experiments, two methods have its own advantages regarding the stages of the experiment, for instance when the participants were with their clothing and when temperature need to be continuously registered, it is not possible to take IRT pictures. In this case, thermal contact sensors had some advantages.

Quesada et al. [16] conducted another study on relationship between skin temperature and muscle activation during cycling. Thermographic data collection and analyses were made with FLIR E60 from the surfaces of each participant’s legs while they were in standing position. The thermal images of ten participants’ legs were recorded before the cycling and 10 min after the cycling. The authors observed significant relationship between skin temperature and neuromuscular activation. After cycling, participants who showed larger overall activation and reduced low frequency component for vastus lateralis activation had a better adaptive response of their thermoregulatory system.

Chudecka and Lubkowska [17] used thermal imaging tools to reveal the thermal maps of young men and women also for medicine, physiotherapy and sport. The authors established temperature ranges and distribution on the body surfaces of men and women. Each thermogram of each participant was recorded by ThermaCAM SC500 model infrared camera in the standing position. They took the measurements in the afternoon after 4 pm. In this study, the important parameters were BMI, body surface PBF and body mass.

According to Silva et al. [18], hybrid analysis for dynamic infrared thermography and breast cancer detection was conducted through temperature time series. The measurements were taken quantitatively with FLIR SC620 thermal camera by temperature changes in the body surfaces. As a conclusion, after analyzing and identifying time series, it would be possible to detect abnormality in the suspicious breast region by taking thermal images of the body surfaces.

In the study of Oliveira et al. [19], the diagnosis and grading of the injured ankles were made using infrared thermography. Evaluating the benefit of infrared thermography as a significant diagnosis tool for lesion grades was the main aim of the presented study. For capturing the thermograms, FLIR E60 SC uncooled infrared camera was used with some specifications, which are FPA of 320 × 240, NETD of 0.05 °C at 30 °C, accuracy of ±2% of overall temperature reading, long wavelength (7–13.5 μm) and using a 24 °C lens. As a result, high potential validation of IR thermography for the diagnosis of ankle injuries gave important contribution to the literature as a grading indicator.

Contreras et al. [20], classified plantar thermal changes in the diabetic foot of the participants in a room with a temperature of 20 ± 1 °C. The infrared camera model they used for the acqusitions was FLIR E60. They positioned the infrared camera 1 m away from the feet of the participants while the measurements were being taken. The main contribution of this research was to provide automatic and simple identification of significant thermal changes on a diabetic foot of the participants by a single index (TCI).

Estal et al. [21] observed thermal asymmetries in striking combat sports athletes using infrared thermography. Investigating the thermal profiles and asymmetries of Muay Thai athletes in kickboxing was the main aim of the study. The temperatures of lead and rear sides on abdomens were detected through infrared imaging. In conclusion, the authors decided that the asymmetries could be due to repeated strikes and actions in combat. The infrared camera model that they used in their studies was FLIR T335.

According to Habek et al. [22] the brown adipose tissue (BAT) activity after a meal in humans was detected through infrared thermography. Totally 12 participants consisted of male and female joined to the measurements. As an infrared camera model FLIR T-650sc was used to measure skin temperature of the participants. Three hours before the measurements, the participants avodided from stress or any physical activity and did not eat any meal. In conclusion, the authors observed increase in the BAT activity of the male participants and decrease in middle-aged female participants.

Silva et al. [23] evaluated the intraexaminer and interexaminer reliability of infrared images of the plantar surface of the participants with diabetes mellitus. FLIR E60 model infrared camera was used for the photographic records of the thermal images. The findings from the thermal images provided financial support to the clinical evauation processes. The authors also concluded that infrared imaging provided intrareliability and interreliability for temperature measurements of the participants, yet more future studies still need to be conducted for health care evaluation.

In the study of Sarigoz et al. [24] the breast mass was detected through in a pilot study through digital infrared thermal imaging (DITI). The method DITI differentiated the benign lesions from malignant with a great sensitivity, which the DITI had a significant role on it. For the measurements, FLIR ThermaCAM E45 was used in DITI procedures.

In the study of Alpar and Krejcar [25, 26] two different articles in IWBBIO 2017 conference about hand thermography was presented. Alpar and Krejcar used smartphone-based FLIR One to conduct the thermographic surveys. According to the results they obtained, IR thermography is very promising method for revealing hand veins through temperature differences.

However, since SEEK camera types are more suitable for other application areas such as firefighting, animals or buildings in the existing literature, the SEEK infrared camera usage still lacks in biomedical applications. In the study of Peleki and Silva [27] the portable SEEK Thermal Compact XR for the immediate detection of Acute Limb Ischaemia was used. The applicability of the specific thermal camera on the assessment of tissue perfusion was discussed when a 78-year-old woman was in the bed during clinical evaluation of her illness. In conclusion, the presented infrared camera is compatible with many smartphone models and it gave a reference before main evaluations and controls in hospital.

We, in this research, compare the various thermograms of the injured toe of the subject by two different smartphone-based thermal cameras namely FLIR One and SEEK Compact PRO. We apply various coloring formats to the thermal images of the injured toe in order to reveal temperature differences in the thermograms. In the continuation of this research, we will specifically discuss the similarities and differences of FLIR One and SEEK Compact PRO separately. In the following sections, we will present our experiments, which are conducted with two infrared camera types. After the results and discussion part, the conclusion remarks are given in last section of this paper.

### FLIR one and SEEK compact PRO

Regarding the biomedical experiments in this research, we specifically use the infrared camera models explicitly FLIR One and SEEK Compact PRO, which are launched to the market for the labelled applications of Android devices; and the camera models that we benchmark in this study are shown in Table 3. The main differences of the presented thermal cameras with special characteristics are summarized in Table 3. The most significant discrepancy of our thermal camera models are their batteries charging type, which is more user friendly for SEEK Compact PRO since it takes the energy from the Android device; however, FLIR One has its own battery, which necessitates very frequent recharging.

**Table 3:** Comparison of the special characteristics of FLIR One and SEEK Compact PRO

	FLIR One	SEEK Compact PRO
	Android	Android
Operated device	Thermal detector	Thermal detector
Detector types	3rd generation	3rd generation
Generation	8–14 μm	7–14 μm
Infrared spectrums	Smartphone-based compact	Smartphone-based compact
Portability	Own format in a database	JPEG
Image extraction format	343 × 458 portrait	1280 × 720 landscape
Image resolution	Own battery	Battery of the Android device
Battery usage	70mK	70mK
Thermal sensitivity (NETD)	Fixed 15 cm — Infinity	Adjustable
Focal distance	160 × 120	320 × 240
Thermal resolution		

Second difference is the spectrum specialty of FLIR One camera; it is wide but scrambled, which means the spectrum has thermal detector. Under significant illuminance, the image quality of FLIR One is considerably higher than SEEK Compact PRO, yet the images are noisier than SEEK Compact PRO, because they consist of two edges. On that account, when there is an experiment with FLIR One, there should not be direct or dominant light on the object regarding environmental conditions.

The main difference on the usability and technical specifications of two specific camera models is that FLIR One could change the angle of its own camera by its converter, which is particularly significant specialty for some cases especially in biomedicine. The procedure of image extraction is very practical in SEEK Compact PRO, since it is in JPEG format that is stored in a separate folder; yet FLIR One extracts images with its own camera format in a database. However, this characteristic gives some opportunity to alter the Pseudocolor of a captured thermogram despite of its complexity.

We, in this study, focus on only biomedical applications, thus the camera models that we chose for this research provide very promising information of the temperature distribution when a target on the screen is properly touched. The image resolution of the specific cameras are also different from each other; for instance the image format of SEEK Compact PRO is landscape in 1280 × 720 resolution; while the image format of FLIR One is portrait in 343 × 458 resolution. Dynamic contrasting, Pseudocolor portfolio, and plug-n-play simplicity characteristics are quite similar in SEEK Compact PRO and FLIR One.

## Methods

**Fig. 1:**
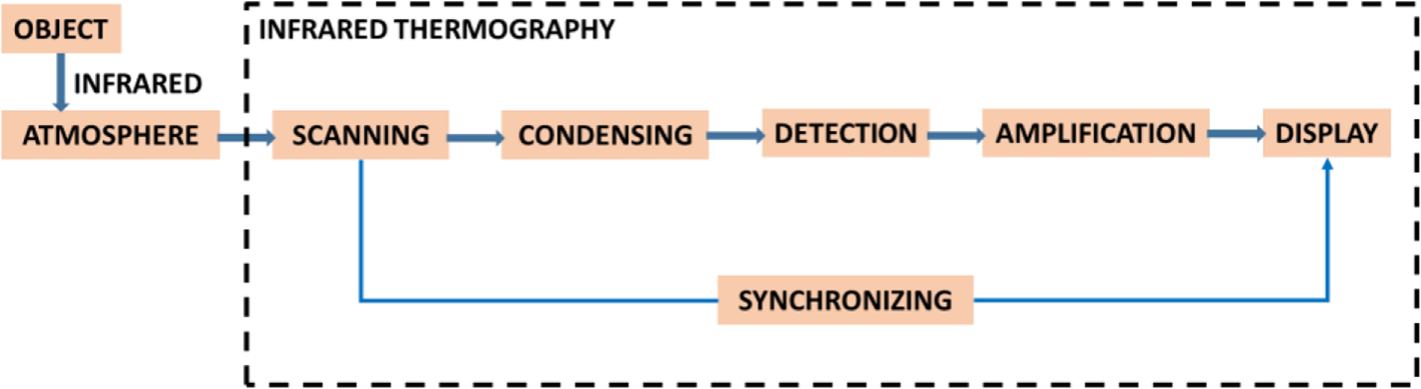
The method of this research

A series of experiments are conducted in the convalescence period of a subject 35/M by SEEK Compact PRO and FLIR One individually under the same illuminance and temperature conditions. For the experiments, an injured toe of the subject without footwear is chosen. The thermal cameras captured the thermograms with original grayscale format and a variety of pseudo colored formats. The explicit dissimilarity in image quality is realized in all thermograms that FLIR One captured the thermal images with higher image quality than SEEK Compact PRO, while the thermograms by FLIR One are noisier than SEEK Compact PRO. The method of this research can be seen in Fig. 1.

### Experiments with SEEK compact PRO

**Fig. 2:**
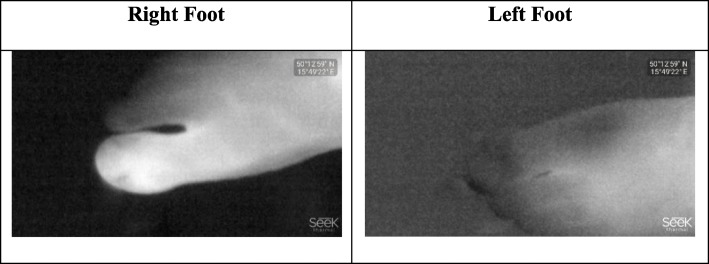
The grayscale thermograms for each foot captured by SEEK Compact PRO

**Fig. 3:**
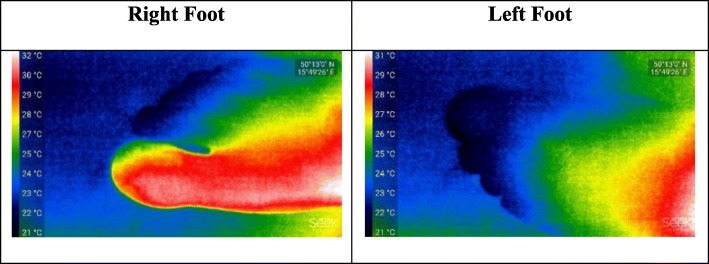
The rainbow pseudo colored for each foot captures by SEEK Compact PRO

**Fig. 4:**
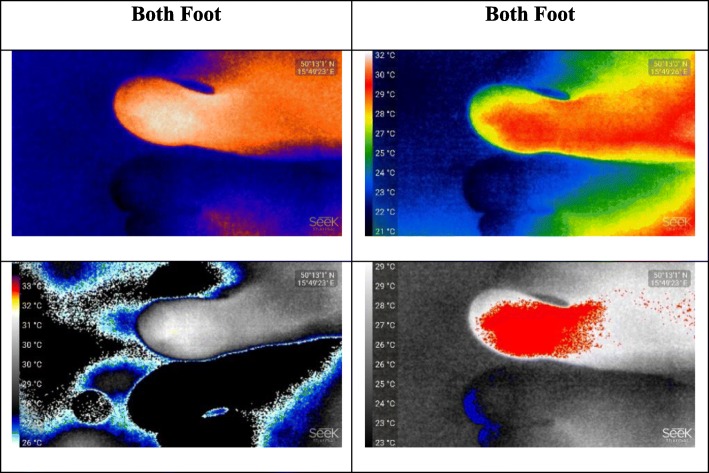
Various pseudocolored thermograms of SEEK Compact PRO for both foot

We, in the first experiment, focus on the toes of the subject independently by extracting grayscale thermal images of the toes. According to Figs. 2, 3 and 4, the thermal images by SEEK Compact PRO are in 1280 × 720 landscape image resolution. If one by one comparison is made, because of dynamic contrasting, an existence of a medical problem on the toe of the subject is not clearly identified. In addition, due to the different background color, right foot seems to be hotter than the left one. The grayscale thermograms individually captured by SEEK Compact PRO for both foot are shown in Fig. 2.

Due to dynamic contrasting in the previous experiment, we chose rainbow Pseudocolor specialty of the camera this time in order to distinguish contrast difference, yet the dynamic contrasting is still recalibrating the hot and cold places of the thermal images on the screen. We separately captured two feet in rainbow Pseudocolor format. According to Fig. 3, red color refers to the hot places in both thermal image and the toe in red color on the right foot represents the injured part.

In order to understand the effect of dynamic contrasting in pseudo colored thermal images, we captured both feet in various formats, thus, the temperature differences are quite visible. In Fig. 4, four different pseudo colored thermal images captured by SEEK Compact PRO for both foot is shown. In the lowermost part of Fig. 4, both format type in the thermal images originally exists in the SEEK Compact PRO, and they do not exist in FLIR One camera type.

### Experiments with FLIR one

**Fig. 5:**
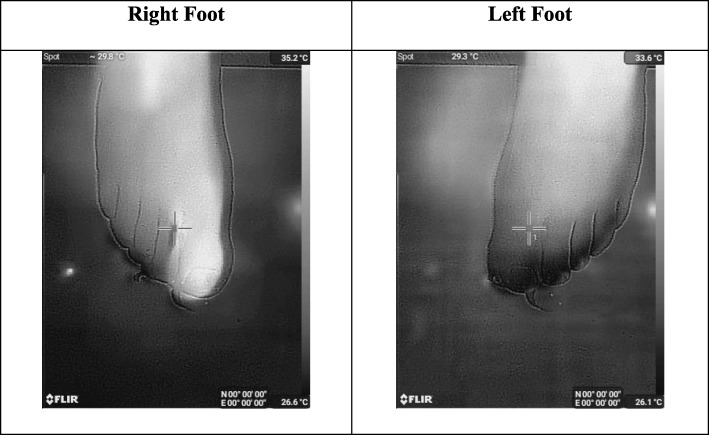
The grayscale thermograms for each foot captured by FLIR One

**Fig. 6:**
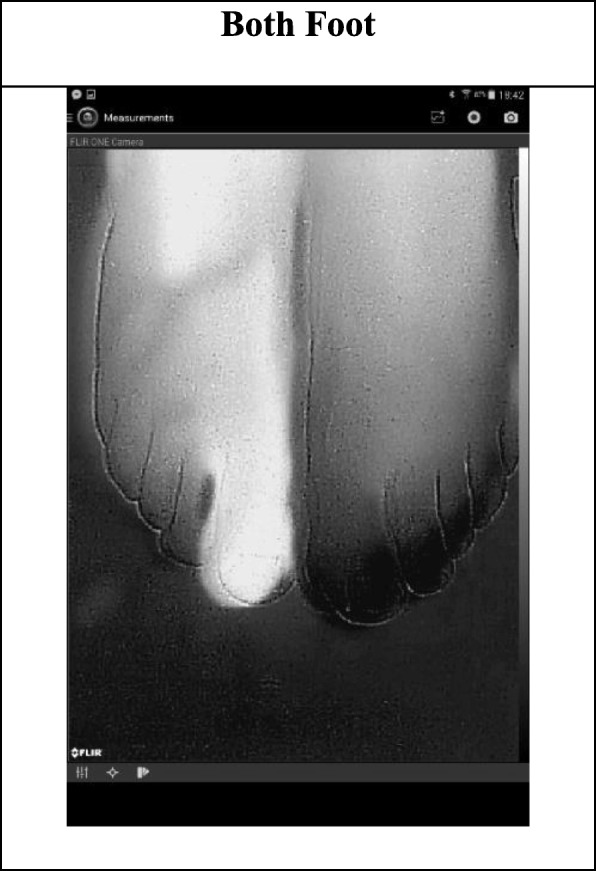
The grayscale thermogram for both foot captured by FLIR One

We carried out the second experiment with FLIR One camera under the same light and temperature conditions and on the same toe of the same subject both one by one and together. The thermal images captured by FLIR One are in 343 × 458 portrait format and noisier than SEEK Compact PRO. Fig. 5 presents the separate grayscale thermal images of right and left foot. However, the comparison made by individually is again less convincing than the comparison made by simultaneously. On the other hand, in terms of temperature differences by color contrast, we can easily recognize the medical problem in grayscale thermal images in Fig. 6, which are captured together.

**Fig. 7:**
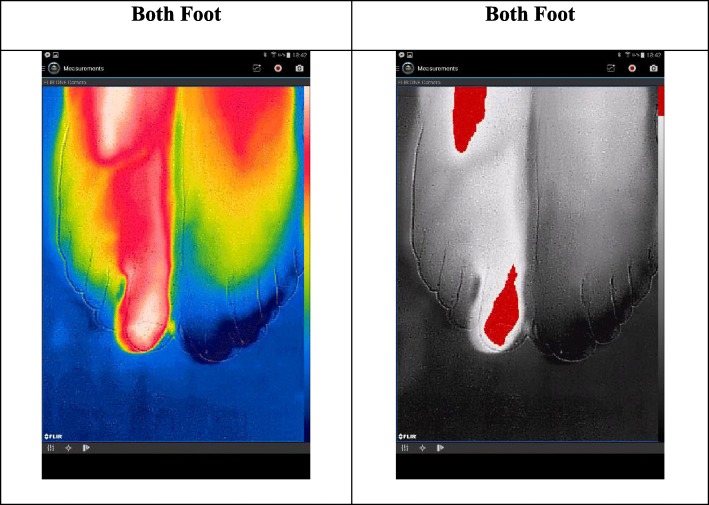
Various pseudo colored thermograms of FLIR One for both foot

In order to indicate temperature difference clearly, we intentionally captured two feet together in original pseudocolored thermograms specialty of FLIR One in Fig. 7. Because the both foot are in different color in both thermal images, the injured toe in the right foot is easily identified. Moreover, since they refer to the cold parts, all toes in the left foot are in blue and gray; yet hot parts are in red color. We clearly see the contours in all thermograms belong to FLIR One camera and this specialty is only exist in FLIR One camera, not in SEEK Compact PRO.

## Results and discussion

We, in this paper, use two different smartphone-based infrared camera models namely FLIR One and SEEK Compact PRO to obtain thermal images of the injured toe of the subject. We also apply various formats to the thermograms by comparing with the left toes of the subject. For our biomedical case, both SEEK Compact PRO and FLIR One are very promising thermal camera types for this kind of close-up shots. Except that, and according to the figures in the experiment sections, SEEK Compact PRO is considerably better than FLIR One in close-up shots. On the other hand, detecting the medical problem on the injured toe of the right foot with SEEK Compact PRO is not satisfied enough using one by one dynamic filtering in grayscale format. Additionally, due to convalescence period of the subject, the contrast difference between background and the subject is not clearly identified, yet we can easily perceive hot and cold regions with dynamic contrasting after applying rainbow pseudocolor formats to the one by one thermograms. Moreover, in order to detect contrast difference more, both foot are captured together in various pseudocolored thermograms of SEEK Compact PRO.

In other respects, we can easily realize the contrast difference between background and the subject in FLIR One camera type, since it has high image quality and visibility of the subject contours, even images are noisier than SEEK Compact PRO. We also use various pseudocolored formats in thermal images of FLIR One camera to compare the performances of the formats. To show temperature differences, we intentionally capture various pseudocolored thermograms of FLIR One, which originally belong to this camera type.

Furthermore, since the thermal sensitivity of two thermal camera models are the same which is 70mK, both cameras can distinguish little temperature differences on the scene at the same level, thus the Noise Equivalent Temperature Difference in other words NETD is the same for both FLIR One and SEEK Compact PRO. It can be also said that electronic noise rating of the system is the same for both thermal camera types. When the noise is same with the smallest measurable temperature difference, it means the detector has already reached its limit of its ability to resolve a useful thermal signal. The more noise means that the NETD value of the detector is higher.

In addition to above, there are some differences between fixed focal distance thermal cameras and adjustable ones. In our research, we are using FLIR One as fixed focal distance and SEEK Compact PRO as adjustable focal distance. The fixed focal distance cameras tend to result in low resolution images and they are used for targets from about 45 cm and further. Besides, thermal resolutions are different for each thermal camera types, which is 160 × 120 for FLIR One and 320 × 240 for SEEK Compact PRO. The higher the thermal resolution is, the more image clarity and sensitivity can be seen.

### Suggestions and new possibilities

Given the technological developments and new perspectives for medical problems, utilization of IR cameras should be enhanced in biomedicine and they would be primary alternative for disease detection in human body. Moreover, the significance of novel methodologies for IR cameras would be highlighted in future studies to be conducted for biomedical problems. Recent advances should shed light on future contributions and opportunities for scientific community that are interested in IR thermal camera applications in biomedicine. Therefore, future suggestions and projections are listed as below:

- In order to find invisible problems faster than ever, there are new generation thermal cameras such as FLIR One PRO [8] have come into the market, thus future contributions will be made with these cameras for biomedical purposes.
- Since the FLIR One PRO [8] is enhanced with the more advanced image processing methods, these new series thermal cameras offer 4 times bigger native resolution and sharper image clarity when they are compared to FLIR One model.
- FLIR Tools Apps [8] provide live stream video when they work with mobile devices; this specialty allows us to monitor from a safe distance and offer opportunity to show others what is going on.
- While making IRT detection for human body diseases, for each part of the body, more accurate data should be collected with thermal cameras for the detection of biomedical problems in the preparation process.
- In the recent literature, there are image processing methods rather than video processing, thus in order to recognize changes in the temperature, capturing thermal videos would be useful for the inspection of diseases.
- Recent progresses should be followed up regularly, since the thermal camera technology is dynamically growing day by day.
- Even subsequent to little injuries in any part of human body such as head, arms or legs, damages should be monitored with IR thermal cameras.
- The IR cameras consist of photon detectors in combination with thermal detectors should be preferred when the visual representation of the investigated diseases matters; otherwise, the IR cameras only with thermal detectors could be recommended as well.
- The smartphone-based IR thermal cameras could be used for small disease detection in human body at home. Because, they are mostly compatible with IOS and Android devices.
- The current reports on biomedical researches should also be followed up regularly for the invention and application of more technological IR thermal cameras.
- The IR cameras with new detector arrays or more complex detectors could be integrated to the scanning systems in order to detect more challenging biomedical problems in human body.

According to the results of our research study, the awareness on the use of IR cameras in biomedical problems should be increased and the most challenging human body diseases would be more accurately identified by combining various methodologies and filtering methods in IR thermography.

## Conclusion

Standard thermometers and conventional camera types are not satisfying thermal detection in many application areas especially in biomedicine towards improvements in technology and science, which necessitate the use of IR cameras. Among the application areas of IR thermography detection, transportation safety, building diagnostics, fire safety enhancement and biomedicine could be found mostly in the existing literature. Additionally, more precise and non-invasive observation of medical problems is possible with IR cameras rather than conventional camera types. The unique advantage of the IR technique in biomedicine is that, the subject is not exposed to unsafe radiation during the clinical assessment of the patients. More practical and faster analysis of even simple medical problems is also possible with IR techniques in biomedicine.

Providing various thermal images of a biomedical problem by making quick and comparative analysis with two specific smartphone-based infrared cameras is the main contribution of this study. We also show the effectiveness of the presented method for the injured toe of the subject in his convalescence period. For future study, more suitable IR camera types could be preferred for an ideal and noticeable biomedical detection, according to our comparative analyses with the presented camera models in the previous sections of this research. Since our presented methodology is closer to qualitative biomedical diagnosis, more quantitative future experiments should be conducted to explore high potentiality of the infrared cameras for the biomedical problems.
